# HIV-, HBV-, and HCV-Related Information on U.S. Fertility Clinic Websites: A Content Analysis

**DOI:** 10.1089/heq.2019.0100

**Published:** 2020-08-14

**Authors:** Margot Kelly-Hedrick, Marielle S. Gross

**Affiliations:** ^1^ Department of Psychiatry and Behavioral Sciences, Johns Hopkins University School of Medicine, Baltimore, Maryland, USA.; ^2^ Berman Institute of Bioethics, Johns Hopkins University, Baltimore, Maryland, USA.

**Keywords:** human immunodeficiency virus, hepatitis B virus, hepatitis C virus, infertility treatment, health equity, health care access

## Abstract

**Purpose:** People living with human immunodeficiency virus, hepatitis B virus, or hepatitis C virus (PLHIV/HBV/HCV) face barriers to assisted reproductive technologies (ART), in part, due to laws and professional regulations mandating dedicated laboratory facilities and storage tanks for reproductive tissue to minimize theoretical risk of cross-contamination. These guidelines greatly increase the expense of providing equal care, however, fertility clinics are neither required to treat nor disclose whether they treat PLHIV/HBV/HCV. Clinics' websites are an important source of information regarding available services for prospective patients and referring providers. We assessed whether clinic websites disclose availability of ART for PLHIV/HBV/HCV.

**Methods:** Websites for Society for Assisted Reproductive Technology-accredited clinics in Northeast and South Atlantic United States were searched systematically for HIV-, HBV-, or HCV-specific content. Qualitative and thematic analysis was performed. Clinic characteristics (annual volume, practice setting) were collected.

**Results:** Of 136 websites, nine (6.6%) had information relevant to PLHIV seeking infertility treatment, and seven (5.1%) offered at least some treatments. Three clinics (2.2%) also mentioned treatment information relevant for PLHBV/HCV, one of which offered treatment. Information was often difficult to find or interpret. By contrast, 77/136 (56.6%) of clinics mentioned universally screening patients for HIV and 77/136 (56.6%) mentioned screening for HBV/HCV before ART.

**Conclusion:** Given economic disincentives to providing ART to PLHIV/HBV/HCV under current guidelines, the paucity of clinics openly offering treatment suggests a troubling lack of transparency or, possibly, a lack of available care. Further research should examine the impact of current guidelines and whether dedicated facilities and storage are medically indicated.

## Introduction

Assisted reproductive technologies (ART) (e.g., *in vitro* fertilization [IVF]) offer the ∼12–15% of U.S. couples struggling with infertility, and many others with social infertility (e.g., some in the lesbian, gay, bisexual, transgender, and queer or questioning (LGBTQ) community, single individuals), the opportunity to have a biologically related child.^1–3^ Unfortunately, most health insurance companies do not cover infertility treatment, causing severe economic disparities in access.^4^ Geography, race, marital status, and sexual orientation also impact access to ART, raising further concerns of distributive justice.^4–8^ ART is particularly constrained for people living with the human immunodeficiency virus and hepatitis B or C virus (PLHIV/HBV/HCV) by virtue of existing laboratory and professional regulations, despite the established safety, efficacy, and both legal and ethical arguments favoring equal access.^9–11^

Federal law for human cellular and tissue products and recommendations by the American Society for Reproductive Medicine (ASRM) mandate *separate facilities and storage dedicated for reproductive tissue from PLHIV/HBV/HCV due to theoretical risks of cross-contamination* (Table 1).^12,13^ This greatly increases the expense of providing equal care, although its benefits are not proven.^12^ As per the ASRM, ART should not be withheld from PLHIV/HBV/HCV, however, individual clinics may either *treat or refer* and public reporting of practices is not required.^14^ The financial disincentives for dedicated facilities and storage may be reinforced by disproportionate socioeconomic disadvantages among PLHIV/HBV/HCV.^15,16^ The increased biological infertility among PLHIV/HBV/HCV exacerbates this inequity.^17^

**Table 1.** Legal Regulations and Professional Guidelines Pertaining to Facility Setup for Reproductive Tissue from People Living with Human Immunodeficiency Virus, Hepatitis B Virus, or Hepatitis C Virus

Source	Excerpt	Comment
Food & Drug Administration, U.S. Department of Health & Human Services Code of Federal Regulations Title 21, Chapter I, Subchapter L, Part 1271, 2017 Regulation of Human Cells, Tissues, and Cellular and Tissue-Based Products (HCT/P)^13^	“You must divide a facility used in the manufacture of HCT/Ps into separate or defined areas of adequate size for each operation that takes place in the facility, or you must establish and maintain other control systems to prevent improper labeling, mix-ups, contamination, cross-contamination, and accidental exposure of HCT/Ps to communicable disease agents.” §1271.190	These regulations mandate how human cells, tissues, and associated products should be handled. The excerpt provides instructions on how facilities should have separate areas for different operations to prevent transmission of infectious diseases, both to other samples and to laboratory personnel, among other consequences such as mix-ups and incorrect labeling of samples.
ASRM, 2013 Recommendations for Reducing the Risk of Viral Transmission during Fertility Treatment with the Use of Autologous Gametes^12^	“Although there is no documentation of cross contamination of stored human tissue, it is highly recommended that samples from viral carriers be processed in a separate laboratory or designated space within the main laboratory”	The ASRM's professional guidelines provide interpretation of the federal laws in the contexts of fertility facilities/laboratory settings. In this excerpt, they propose the so-called dual setup that tissue from people living with viral infections should be handled in a separate laboratory facility. These guidelines emphasize HIV, HBV, and HCV as these are the viral infections most frequently seen in care.^12^

ASRM, American Society for Reproductive Medicine; HBV, hepatitis B virus; HCT/P, human cells, tissues, and cellular- and tissue-based products; HCV, hepatitis C virus; HIV, human immunodeficiency virus.

**FIG. 1.**
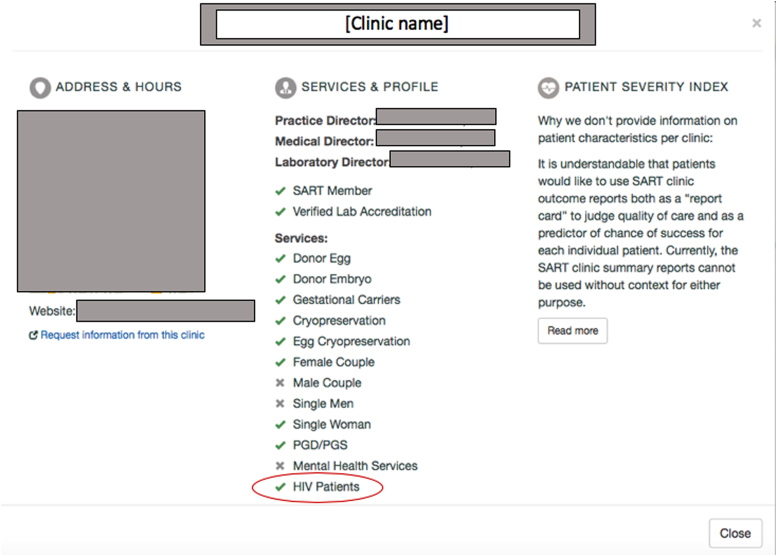
Screenshot of a clinic's profile from the “Find a Clinic” database by the Society for Assisted Reproductive Technology. The *gray boxes* have been added to protect the privacy of the clinic. *Red circle* has been added to identify the “HIV patients” field. HIV, human immunodeficiency virus.

The ASRM estimates that <3% of U.S. clinics treat PLHIV (and presumably PLHBV/HCV, as regulations apply similarly), although specific clinics offering treatment are not identified.^14,18^ The lack of a reliable referral system has spurred calls for increased transparency from patients and referring providers.^14,19^ In response, the Society for Assisted Reproductive Technology (SART) augmented the clinic profiles on their national database^†^ in mid-2019 to include whether services are provided to “HIV patients” (Fig. 1). Although this addition signals progress, barriers persist, as PLHBV/HCV are not addressed, and whether processing and storage of reproductive tissue are available, as opposed to preliminary workup and other medical or surgical treatments, is not specified. More information and transparency are still needed.

The majority of prospective fertility patients go online for information about clinics and their services.^20^ Studies have examined fertility clinic website content as a possible indication of clinic values and the patient populations they treat.^21,22^ We conducted a content analysis of fertility clinic websites to determine if they treat PLHIV/HBV/HCV.

## Methods

A content analysis and qualitative thematic analysis of fertility clinic website content related to HIV/HBV/HCV were conducted from July 2017 to April 2019. The study was deemed exempt by the Johns Hopkins Medicine Institutional Review Board.

### Sample

SART-accredited clinics from the Northeast and South Atlantic regions were selected as a convenience sample with a relatively high prevalence of HIV/HBV/HCV.^23–25^

In July 2017, a list of clinics and their websites was compiled from SART's “Find a Clinic” database and was routinely updated throughout the data collection process. For nonfunctional URLs, a Google search identified the correct site. Duplicate websites were analyzed only once (i.e., satellite locations of large practice groups).

Seventeen states and the District of Columbia were included. The number of SART-approved clinics per state ranged from one (WV and RI) to 31 (NY). One state (NH) had no SART-approved clinics. Another state (ME) has only one SART-member clinic, which was a satellite location of a group based in MA. One clinic had no website and one website was out of service at the time of coding; both were excluded from analysis.

### Clinic information

The number of cycles per year and the clinic location (state and zip code) were collected from the SART database. Clinics were also identified as private, academic/university-affiliated, or government-affiliated.

### Codebook

Websites were coded for treatment-specific screening and alternative content related to HIV and HBV/HCV (Table 2).

**Table 2.** Codebook for Content Analysis of Human Immunodeficiency Virus- or Hepatitis B Virus/Hepatitis C Virus-Related Content on Fertility Clinic Websites

Code	Definition
Treatment-specific content	Any information related to infertility treatment for PLHIV or PLHBV/HCV (e.g., stating the fertility clinic offered or did not offer specific services to individuals in these populations)
Screening content	Information related to screening for HIV, HBV or HCV (e.g., testing, medical history, exposure, vaccination)
Alternative content	Content that did not fit into the first two categories (e.g., privacy policies, legal information, pathophysiology).

PLHBV/HCV, people living with HBV/HCV; PLHIV, people living with HIV.

### Coding procedure

All pages on each website were manually searched for “HIV,” “Human Immunodeficiency,” “Hep,” and “Hepatitis,” using the find function (“CTRL-F”), and embedded search bars. For a subset of 40 websites, all available PDF and Word documents were downloaded and searched and relevant findings from these documents were recorded. For each website, the number of mentions of each keyword for each content type was recorded based on surrounding context. This was later collapsed into any mention versus no mention for HIV and HBV/HCV for each content category. Two coders performed these website reviews, and intercoder consistency was evaluated between the coders by an additional member of the research team (M.S.G.).

### Analysis

For treatment-specific content, the mention and surrounding text were copied and read by two members of the research team to elucidate themes and key takeaways. Illustrative quotes for screening and alternative content were also recorded. Descriptive statistics of clinic characteristics and codes were calculated.

## Results

### Clinic characteristics

Of 136 websites, 108 (79.4%) were private, 27 (19.9%) were university- or academic-associated, and 1 (0.7%) was government-affiliated. The median number of cycles per year was 379 (interquartile range=512).

### Treatment information for PLHIV/HBV/HCV

Nine (6.6%) of the websites had information relevant to infertility treatment for PLHIV; 7 (5.1%) provided at least some treatments, 1 did not treat, and in 1 case it was unclear whether treatment was available. Three (2.2%) had information pertaining specifically to PLHBV/HCV, but only one (0.7%) clinic clearly offered treatment.

The information was found in various places on the websites, including pages about LGBTQ treatment options, diagnostic testing, donation, patient resources, and, in two cases, buried in dense PDF documents intended for new patients to download/complete before their appointment. The information was often vague, and it was difficult to discern which services, if any, were offered for PLHIV/HBV/HCV.

#### Providing treatment for men

Seven clinics mentioned that reproductive treatment for men with HIV was available; one of these clinics also mentioned treatment for men with HBV/HCV. One clinic reported having “worked successfully with over 25 HIV-positive dads who as a result have achieved their goal of healthy parenthood” and another welcomed “discordant HIV couples (where the man is HIV positive but the woman is not).” Upon reviewing the websites with treatment-specific content after the coding period, we discovered the latter clinic had since closed.

Some clinics clearly stated that they offered IVF with sperm washing techniques:

“[Clinic 5] utilizes the Bedford Spar/HIV protocol to treat affected men seeking to create biologically related embryos… The Special Program of Assisted Reproduction (SPAR) is a world renowned program that combines PCR HIV Semen Testing and Sperm Washing to screen and prepare semen specimens from HIV-infected men for safe IVF procedures.”

In other cases, it was unclear if IVF was available for men with HIV:

“Gay men who are HIV-positive can father children, because the HIV virus cannot infect sperm. But, the semen or surrounding fluid may be infected. That is one reason we use sperm washing to purify the sperm.” (Clinic 126)

The website did not note whether washed sperm could be used for IUI, IVF, or stored long term (cryopreservation).

When discussing sperm cryopreservation, one clinic stated that the patients would have to first undergo a blood test, and if test results were abnormal (i.e., HIV detected) they would require “special arrangements… to be made at the long-term storage facility” (Clinic 22), without specifying whether the patients would incur an additional cost. The clinic also wrote:

It is important that diseases such as Hepatitis and HIV be ruled out before obtaining and storing the tissue. If this blood work cannot be obtained before cryopreservation, the tissue will be placed in quarantine while frozen pending the results. If Hepatitis and/or HIV tests are positive, [clinic 22] *reserves the right to thaw and destroy the tissue*. Long-term storage facilities will often not store potentially contaminated tissue.” (Clinic 22, emphasis added)

This seemingly conflicting information is difficult for patients to interpret; patients may be responsible for identifying a facility willing to store their reproductive tissue.

#### Providing treatment for women

Four websites included treatment information for women with HIV; three clearly offered some services, two of which detailed which specific services are available. Another clinic spoke broadly about infectious diseases; however, it was unclear if they offered long-term storage:

“If either partner is positive, your sperm, eggs and/or embryos can be frozen, but only with other sperm and embryos that may be infected with similar contagious diseases. These tests are all required to be repeated annually. Not everyone is required to have all of these tests. Some are only required of people using either donor eggs or donor sperm.” (Clinic 44)

This clinic's testing for donor recipients may be for documenting a patient's disease status before implanting donor's tissue, guarding against potential claims that donor tissue was responsible for transmission. This clinic also wrote: “a woman intending to carry a pregnancy who is positive for Hepatitis B surface antigen will need a letter of medical clearance” (Clinic 44), indirectly suggesting that they treat women with HBV.

The final clinic provided specific information about treatment for women with HIV:

“If the female recipient is found to be HIV positive before treatment, she should be referred to an appropriate infectious disease specialist for counseling on issues concerning HIV disease… A positive HIV-1 or -2 test of the female recipient should not be used as an exclusionary criterion for treatment with DI [donor insemination]” (Clinic 122)

The document later says:

“[Clinic name] does not perform inseminations using sperm obtained from men testing positive for HIV, Hepatitis B, or Hepatitis C. Couples in which one or both partners test positive for HIV, Hepatitis B or C should be treated by fertility centers having the appropriate laboratory resources.” (Clinic 122)

The site provided no information on referrals to treating clinics.

#### Unclear treatment information content

On two websites, we were unable to discern if the clinics provide treatment for PLHIV/HBV/HCV. A downloaded document from one wrote, “a donated Embryo Recipient who already has Hepatitis may receive donated embryos from Donor Parents with Hepatitis” (Clinic 22). It was not explicitly stated whether the respective clinic would perform the procedure. The other clinic was also unclear, stating in its nondiscrimination policy:

“[Clinic 108] complies with…the Civil Rights Act of 1964…and…the Rehabilitation Act of 1973…so that no person shall, on the grounds of race, color, religious creed, ancestry, union membership, age, gender, sexual orientation, gender identity or expression, national origin, *AIDS or HIV status* or disability…be excluded from participation in, be denied benefits of, or otherwise be subjected to discrimination in the provision of any care or service.” (Clinic 108, emphasis added)

Notably, when we revisited this website at the end of the coding period, the statement had been amended to no longer include mention of “AIDS or HIV status.”

### HIV/HBV/HCV screening content

Information about screening patients for both HIV and HBV/HCV was included on 77 (56.6%) of the websites. Frequently, websites stated that preprocedure testing would be required for both patients and donors, suggesting the importance of diagnosing these conditions to infertility treatment. While many clinics did not elaborate on why such testing was required, some did:

“Women who have STIs, HIV, or Hepatitis will have a difficult time becoming pregnant and can be more susceptible for miscarriages. If the mother tests positive for either disease, there's a significant chance of passing the diseases to the baby. That's why these types of screenings are essential in your evaluation.” (Clinic 98)

Some clinics also specifically asked about HIV/HBV/HCV, recent exposure to viruses in general, and vaccination records (HBV), when assessing patient or donor medical history, often without specifying how this information would impact a person's ability to donate tissue or receive care.

### Alternative contexts of HIV/HBV/HCV-related content

Of the 136 websites, 36 (26.5%) mentioned HIV and 12 (8.8%) mentioned HBV/HCV in contexts unrelated to treatment or screening.

#### Alternative contents related to HIV

Of the alternative contents, HIV was most frequently mentioned in clinic's privacy policies (e.g., regarding sharing HIV-related information via e-mail) and private health information forms. Informed consent forms from one website included details about inherent risk of viral infection from blood and tissue-based products used during treatment:

“Human serum albumin, a commercially prepared blood product for clinical laboratory use, is added to the egg collection fluid, micromanipulation, and semen preparation fluids. Careful screening is done by the manufacturers to reduce the likelihood of transmission of infectious diseases such as HIV, Hepatitis B and C. To date there have been no documented cases of disease transmission linked to human serum albumin usage at [Clinic 55]. We [the patient(s)] understand and accept the remote risk that use of these blood products could result in the transmission of HIV and Hepatitis.” (Clinic 55)

This legalese reflects concerns about HIV/HBV/HCV infection during treatment.

Four clinic websites cited a specific study,^‡^ “The psychological impact of infertility: a comparison with patients with other medical conditions,” in which the stress of living with infertility was comparable with the stress of living with HIV.^26^ For example, one website posted a blog entry about coping with infertility on Mother's day:

“Infertility can lead to feelings of sadness, anger, insecurity, and fear. It's normal to have these feelings, and it's important to allow yourself to feel these emotions. Research has shown that the level of emotional distress associated with receiving a diagnosis of infertility is comparable to the distress associated with a diagnosis of HIV or cancer.” (Clinic 51)

This content addresses patients with infertility broadly speaking, as opposed to PLHIV who themselves are struggling with infertility.

HIV was also mentioned as a cause of infertility, on three websites' glossary of terms, three physicians' biographies (e.g., research interests), and on one website discussing general information on sexually transmitted diseases. Other mentions occurred in the blog sections of websites, including scientific articles on HIV, posting about a charity event for PLHIV, and a post about annual physical checkups. Two clinics mentioned HIV when discussing the Zika virus. One wrote:

“We make assumptions, based on precedent, that sperm washing (as we do with IUI or IVF) will prevent or significantly reduce egg exposure to virus. Again, there is precedent as these are the same protocols that are followed when a man has other communicable viruses, Hepatitis C and HIV, for instance. Sperm is washed and isolated, concentrating viable sperm and removing the elements of semen that are unwanted.” (Clinic 42)

#### Alternative contents related to HBV/HCV

The alternative contents related to HBV/HCV were varied, although they largely mirrored the alternative mentions of HIV regarding release of private health information, causes of infertility, glossary of terms, and reference to Zika virus. HBV was also mentioned in the context of vaccination recommendations. Chronic hepatitis was mentioned as a potential result of untreated pelvic inflammatory disease. One clinic's blog mentioned intralipid infusion, an immunotherapy for women with recurrent miscarriages, and cautioned against using this therapy in patients with kidney or liver diseases such as hepatitis.

## Discussion

This study surveyed clinic websites from the perspective of prospective patients or referring physicians seeking ART services. Our content analysis of fertility clinic websites in the Northeast and South Atlantic regions found scant information relevant to PLHIV/HBV/HCV. While some clinics that treat PLHIV/HBV/HCV may not disclose this on their website, the lack of information about treatment availability may create a barrier as patients are unable to tell whether a clinic will provide them with services, particularly given the context of laboratory restrictions and reported scarcity (<3%) of treating clinics.^14,18^

### Treatment information for PLHIV/HBV/HCV

Where it existed, the treatment-related information on websites mostly pertained to PLHIV and rarely PLHBV/HCV. While a few websites provided clear clinical information, most seemed more focused on legal and professional guidelines. Clinically actionable information regarding services offered, potential requirements for treatment (e.g., undetectable viral load), and details relevant to serodiscordant/seroconcordant couples were largely absent.

One clinic referred to “special arrangements” for long-term storage in language that suggested additional costs for the patient. In *Bragdon v Abbott* (1998), a woman sued her dentist over extra charges incurred because he required her to have the procedure in the hospital due to theoretical risks of cross-contamination.^27^ The Supreme Court ruled that providers must prove that measures beyond universal precautions are medically necessary.^27^

PLHIV have successfully sued health care providers for denying equal access to services due to similar theoretical cross-contamination risks in other health care settings, with some court rulings requiring publicly posted nondiscrimination statements.^28,29^ Critically, separate facilities and storage tanks have not been proven as medically necessary for ART.^12^ Whether the extra costs of adhering to guidelines are passed onto patients is unknown, although the website content above and anecdotal evidence suggest this possibility in some cases.

Another clinic stated they “reserve the right to destroy tissue” if individuals test positive for HIV/HBV/HCV following collection. To those aware of laboratory guidelines, this implies a lack of dedicated storage facility space. To prospective patients with previously established diagnoses, this policy expresses disregard for individuals' time, effort, expense, and discomfort of undergoing tissue harvesting procedures and violates the sanctity otherwise assigned to reproductive tissue (like embryos). As concerns for cross-contamination are theoretical and third-party storage facilities exist, this approach is inappropriately punitive and appears to be informed by fear and stigma.^12^

### HIV/HBV/HCV screening content

Nearly 60% of websites disclosed *screening for HIV/HBV/HCV*, as per ASRM recommendations.^12^ Screening-related content was for all-comers and asserts medical significance of diagnosing these conditions before ART. Mentions of screening practices did not elucidate how results impact access to treatment, but rather seemed to inform and reassure prospective patients.

The legal mandate to screen tissue donors for HIV/HBV/HCV was also frequently mentioned without disclosing how these conditions impact donor eligibility.^30^ Broader inequities in blood, organ, and tissue donation, specifically in relation to risk factors for HIV/HBV/HCV, are increasingly recognized.^31,32^ In this context, extending screening to all ART patients, although only legally required for third-party donors (who may be unknown to the recipient), may unintentionally increase barriers to care under existing laboratory guidelines. Further investigation of who is meant to benefit from a universal required screening and unintended consequences is warranted.

### Alternative contexts of HIV/HBV/HCV-related content

The alternative content relevant to PLHIV/HBV/HCV was primarily related to privacy policies. Overall, other alternative mentions alluded to stigma and anxiety regarding infectious disease, especially HIV, in the assisted reproduction community.

### Limitations and future directions

Our sample covered geographically, socioeconomically, and racially diverse regions with high prevalence of HIV/HBV/HCV, although it was limited to the Northeast and South Atlantic SART-approved clinics and may not represent other U.S. regions. Importantly, we achieved saturation as no new findings emerged when subsequent states were added. We find that few clinics report offering care to PLHIV/HBV/HCV on their websites; however, it is possible that some of these clinics offer treatment without disclosing this on their website. We did not assess how website content impacts patients' attitudes and behaviors about seeking care.

Our study occurred during a period where the culture surrounding fertility care for PLHIV/HBV/HCV is in flux.^33^ In mid-2019, after website analysis was complete, the “HIV patients” field was added to the SART “Find-a-Clinic” database (Fig. 1), suggesting an encouraging culture shift toward increased transparency. Upon accessing every clinic profile, we found that only 5 (1.5%) indicated treatment for PLHIV as of September 2019. We also noted concurrent discrepancies between some clinic profile statuses and respective website content, with both SART clinic profiles indicating treatment without corresponding information on clinic websites and treatment information on websites with no services offered as per the SART profile.

By contrast, a 2018 “secret shopper” survey found that 63% of clinics offered some, although not necessarily all, treatments to a hypothetical serodiscordant couple, and prospective patients received vastly different information about availability of services than referring providers.^19^ This discordance may reflect the variation in which services clinics offer for these populations. The database update does not address the needs of PLHBV/HCV, although they outnumber PLHIV considerably, and are presumably affected equally by laboratory guidelines.

Further research should assess the impact of current policies, limited online information, and universal screening on access to care, as well as the prevalence of PLHIV/HBV/HCV with unmet infertility treatment needs. We should also examine how clinics functionally implement ASRM guidelines and federal law, whose language leaves some room for interpretation; and for those who do not provide care, assess the barriers to treating these populations. Examination of whether such guidelines are truly medically indicated is warranted, especially given the legal precedent that providers must demonstrate insufficiency of universal precautions. Comparison with ART practices in Europe, where less burdensome precautions are used, may be illuminating.^34^

Recent guidelines by Jindal et al. also suggest less burdensome precautions, such as storing tissues using a separate canister within a cryopreservation storage tank, rather than maintaining separate cryopreservation storage tanks.^35^ Further national discussion on laboratory guidelines could help to provide clarity on adhering to regulations and potentially lessen the cost and burden of providing fertility care to PLHIV/HBV/HCV. Exploring these questions is critical, as there are medical, ethical, and legal imperatives to provide equal ART to PLHIV/HBV/HCV.^10^

## Conclusion

U.S. fertility clinic websites largely lack information relevant to PLHIV/HBV/HCV. Increased transparency on clinic websites and national databases may decrease disparities and facilitate referral.

## Abbreviations Used

ART: assisted reproductive technologies
ASRM: American Society for Reproductive Medicine
HBV: hepatitis B virus
HCT/P: human cells, tissues, and cellular- and tissue-based products
HCV: hepatitis C virus
HIV: human immunodeficiency virus
IVF: *in vitro* fertilization
LGBTQ: lesbian, gay, bisexual, transgender, and queer or questioning
PLHBV: people living with HBV
PLHIV: people living with HIV
SART: Society for Assisted Reproductive Technology

## Notes

^†^ https://www.sart.org/clinic-pages/find-a-clinic/

^‡^ Notably there were only 11 women with HIV included in the study, compared with 149 with infertility.
